# Advances in Intestinal-Targeted Release of Phenolic Compounds

**DOI:** 10.3390/nu17162598

**Published:** 2025-08-09

**Authors:** Yunxuan Tang, Wenjing Liu, Jiayan Zhang, Bai Juan, Ying Zhu, Lin Zhu, Yansheng Zhao, Maria Daglia, Xiang Xiao, Yufeng He

**Affiliations:** 1 School of Food and Biological Engineering, Jiangsu University, Zhenjiang 212013, China; tang1834178338@163.com (Y.T.); 18660310376@163.com (W.L.); jiayanzhang1988@163.com (J.Z.); 1000005134@ujs.edu.cn (B.J.); ying307@126.com (Y.Z.); zhu-lin19820402@ujs.edu.cn (L.Z.); zhaoys@ujs.edu.cn (Y.Z.); xiaoxiang1@aliyun.com (X.X.); 2 International Research Center for Food Nutrition and Safety, Jiangsu University, Zhenjiang 212013, China; maria.daglia@unina.it; 3 Department of Pharmacy, University of Naples “Federico II”, Via D. Montesano 49, 80131 Naples, Italy

**Keywords:** phenolic compound, intestinal-targeted delivery, co-encapsulation, wall material, bioavailability

## Abstract

Phenols are natural compounds with considerable bioactivities. However, the low bioavailability and chemical instability of phenols limit their biological functions. This review summarizes recent progress in phenol delivery systems that account for the specific physiological conditions of the gastrointestinal tract. It focuses on the delivery materials for intestinal targeting and the synergistic benefits of co-encapsulating phenols with other functional ingredients. To achieve targeted release of phenols in the digestive tract, factors such as pH, digestive enzymes, and gut microbiota should be fully considered in delivery system designing. Materials like chitosan, sodium alginate, pectin, and guar gum offer effective protection and targeted delivery of phenols due to their pH sensitivity and enzyme-degradable properties. Co-delivery systems that combine phenols with carotenoids or probiotics improve the functional properties of phenols, such as antioxidant activity, anti-inflammatory effect, and regulation of gut microbiota. Probiotics can enhance phenolic compound absorption and probiotic survival in a phenolic–probiotic co-encapsulation system through debonding, bioconversion, and synergistic effects.

## 1. Introduction

As modern lifestyles have changed, the global prevalence of chronic diseases such as obesity, hypertension, and hyperlipidemia has increased significantly [1,2]. For individuals with these conditions, these diseases diminish the living quality and increase the additional burden of medical care. For example, obesity is associated with an increased risk of type 2 diabetes, cardiovascular diseases, stroke, and certain cancers (such as breast, colorectal, endometrial, and pancreatic cancers) [3,4]. Recent studies have highlighted the value of food bioactive compounds to prevent and mitigate the impact of these chronic diseases [5].

Phenols are abundantly present in plants [6]. Research indicates that phenols offer a wide range of health benefits, including antioxidant, anti-inflammatory, lipid-lowering, and gut microbiota-modulating effects [7]. These properties make them effective in preventing various metabolic disorders, such as cardiovascular disease, diabetes, and obesity [7,8,9]. Additionally, phenols can modulate the gut microbiota by promoting beneficial bacteria (such as *Lactobacillus* and *Bifidobacterium*) while inhibiting harmful bacteria (e.g., *Escherichia coli* and *Salmonella*), thus maintaining gut health [10,11,12]. Despite their benefits, phenols face several challenges during the digestion process. For example, phenols can bind to salivary proteases in oral cavity, which causes unpleasant astringent tastes, thereby limiting their palatability in food products [13]. In addition, phenols are chemically unstable and can be degraded under various conditions, resulting in the loss of their bioactive properties. This instability complicates their processing, storage, and transportation [14]. Table 1 provides a comprehensive overview of the bioactive properties and technological challenges associated with commonly consumed phenols, detailing their therapeutic potential and limitations in industrial applications. For instance, tea polyphenols can be degraded when they are exposed to light, high temperatures, or transition metals, undergoing irreversible chemical changes [15]. Furthermore, phenols are prone to instability in the acidic conditions of the stomach, preventing them from reaching the gut in their bioactive form [16]. These challenges not only hinder the bioaccessibility of phenols in the intestine but also restrict their application as plant-based nutritional supplements.

Various technological strategies are applied by researchers to overcome the challenges of phenols such as low bioavailability, instability, and limited effectiveness in the intestine. One promising approach is the intestinal-targeted release technology. This method involves designing specific carrier systems that enable the precise release of phenols within the intestinal tract. It not only addresses the issue of the astringent taste of phenolic compounds but also protects them from degradation by gastric acid and digestive enzymes, thereby prolonging their residence time in the intestinal tract [17]. Due to the complex physiological environments of the digestive system, developing effective phenol delivery systems requires consideration of the distinct conditions in different regions. For instance, the acidic pH and the presence of pepsin in the stomach necessitate that the delivery system materials be resistant to both acid and enzymatic breakdown [18]. The physiological environment of the small intestine must also be considered. The neutral pH and high digestive enzyme activity in the small intestine can challenge the stability of these systems. Therefore, colon-targeted delivery systems are often engineered with additional protective layers, such as stable polysaccharides combined with proteins, providing extra protection against degradation [19]. Additionally, the pH difference between the small intestine and colon can be exploited to develop pH-responsive shells for targeted phenol * delivery to the colon [20]. In recent years, there has been growing interests in co-delivery systems, which combine phenols with other bioactive compounds, such as carotenoids and probiotics. These systems can effectively deliver both phenols and other bioactives. Co-delivery systems are designed by selecting compatible bioactives based on their physicochemical properties (e.g., polarity, hydrophilicity, antioxidant properties) and functional activities (e.g., anti-inflammatory activity, anticancer activity and anti-obesity activity), thus enhancing the stability of the system and generating synergistic effects [21,22,23].

**Table 1:** Comprehensive characteristics of common phenolic compounds. Figures source: Retrieved from PubChem database (https://pubchem.ncbi.nlm.nih.gov) (accessed on 10 May 2025).

Bioactive Compound	Chemical Structure	Sources	Solubility (mg/mL)	Pharmacological Properties	Limitations	References
Quercetin	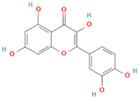	Apples, onions, buckwheat, green tea	0.002	Antioxidant, anti-inflammatory, anticancer, antiviral, antiproliferative, and anti-diabetic in clinical trials	Poor stability under light, heat, and alkaline conditions, poor gastrointestinal stability, and low bioaccessibility (<2%)	[24,25]
Epigallocatechin gallate (EGCG)	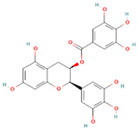	Green tea, black tea, white tea	10	Antioxidant, anti-inflammatory, and anti-colon cancer; efficacy confirmed in vivo in mouse liver injury models	Bitter taste, sensitive to high temperature, oxygen, and pH changes	[26]
Gallic acid	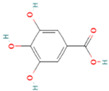	Tea leaves, grapes, berries, gallnuts, mangoes	50	Antioxidant, anti-inflammatory, analgesic, neuroprotective, anticancer, and anti-diabetic in vivo	Astringent taste, large particle size, poor absorption, low bioavailability, and rapid excretion. Unstable at high temperatures	[27,28]
Resveratrol	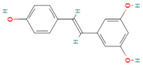	Grape skins, peanuts, polygonum cuspidatum	0.03	Antioxidant, anti-inflammatory, anti-obesity, and anti-colon cancer in vivo	Low water solubility, low oral bioavailability, sensitive to high temperature, oxygen, and light, prone to rapid degradation and inactivation during intestinal metabolism	[29,30]
Ferulic acid	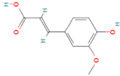	Whole grains, angelica, oats, tomatoes	1	Antioxidant, anti-inflammatory, anti-tumor, and antihyperglycemic activities in vivo	Unstable to oxygen and prone to degradation, poor gastrointestinal stability	[31,32,33,34]
Procyanidin	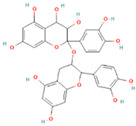	Grape seeds, cocoa beans, apples, berries	0.01–0.05	Anti-obesity, antioxidant, and anti-type 2 diabetes in vivo	Unstable under pH changes, heat, humidity, gastrointestinal environment	[35,36]
Chlorogenic acid	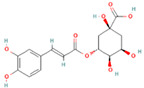	Coffee beans, peaches, plums, apples, eggplants	5–10	Antimicrobial, anti-inflammatory, antioxidant, regulates glucose, lipid metabolism, and antitumor activities in vivo.	Unstable under heat, light, and alkaline conditions	[37,38]
Coumaric acid	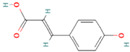	Grapes, olives, coffee beans, tomatoes, carrots, cereals	0.6	Anti-inflammatory, antioxidant, and anti-colon cancer; improve cholesterol metabolism and enhance antioxidant capacity in vivo	Sensitive to pH, temperature, oxygen, light, and enzymes	[39,40]
Curcumin	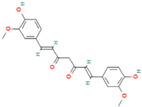	Turmeric, curcuma zedoaria, curcuma aerugionosa	<0.0004	Prevents cancer, cardiovascular diseases, and diabetes, neuroprotective in vivo	Hydrophobic compounds, low bioavailability and high chemical transformation rate, sensitive in alkaline pH (>7), high temperature, and light	[41,42,43]
Anthocyanins	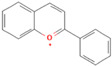	Blueberries, red cabbage, purple, potatoes	10	Antioxidant, anti-inflammatory, anticancer, and anti-diabetic, alleviates chronic intestinal diseases in vivo	Hydrophobic compounds, not resistant to gastric acid, and have low bioavailability	[44,45]
Catechin	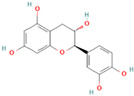	Green tea, black tea, apples, grapes,	10	Antioxidant, antidiabetic, anti-inflammatory anticancer, and antibacterial, inhibit the pathogenesis of colorectal cancers in vivo	Sensitive to pH, temperature, oxygen, and light; instability in the gastrointestinal tract and limited membrane permeability across the intestine	[46,47]

Although existing reviews summarize advances in the preparation of phenol microcapsules, they predominantly concentrate on preparation methods and the design of delivery systems. There is, however, a clear need to investigate the intestinal-targeted release of phenol microcapsules, considering the unique environment of different digestive sites. Furthermore, the co-encapsulation of phenols with other bioactive compounds is gaining attention. This review examines the physiological conditions of the digestive tract and their influence on phenol delivery systems. It also presents wall materials like chitosan, sodium alginate, and pectin, which are ideal for developing phenol-based intestinal-targeted release systems. In addition, this paper also discusses the suitable delivery systems for different water-soluble phenolics as well as advances in the study of phenolics co-encapsulation with probiotics and their interaction.

## 2. Methods

A literature search was performed using the Web of Science, PubMed, and ScienceDirect databases to identify relevant studies published from January 2015 to December 2025. The search was limited to articles published in English in peer-reviewed journals. The following keywords and combinations were used for the search: “phenolic compounds,” “intestinal targeted delivery,” “polyphenol microencapsulation,” “co-encapsulation of polyphenols with probiotics,” and “intestinal-targeted release of polyphenols.” Conference abstracts, non-English articles, duplicate records, and studies not directly related to the intestinal delivery of phenolic compounds were excluded.

The inclusion criteria encompassed original research articles (including in vitro, in vivo, and clinical studies) as well as review articles addressing advances in the targeted delivery of polyphenols and their interaction with gut microbiota or encapsulation techniques. Articles meeting the inclusion criteria were reviewed in full, and relevant references cited within these articles were also screened to ensure comprehensive coverage of the topic. A narrative synthesis of the selected literature was then conducted to summarize the current knowledge and identify gaps for future research.

## 3. Physiologic Environments of Digestive Tract and Their Effects on Phenol Delivery Systems

**Figure 1:**
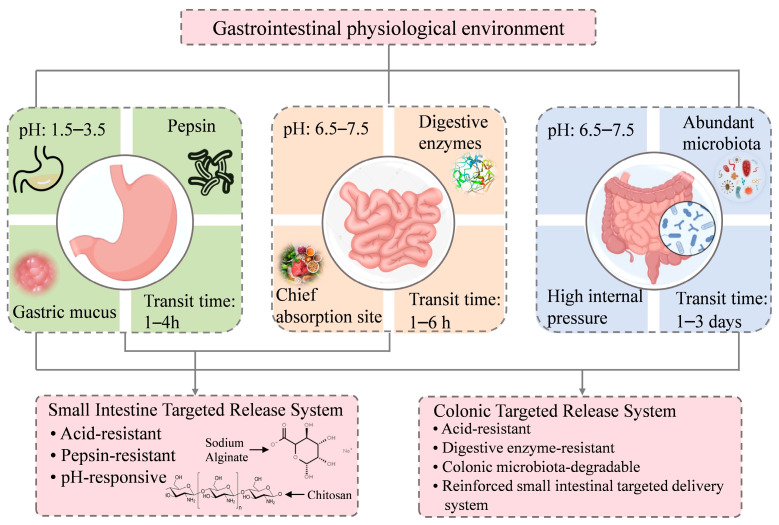
Schematic of the gastrointestinal physiological environment and wall material selection strategies.

When preparing gastrointestinal targeted-release systems, selecting wall materials is extremely important. The characteristics of the gastrointestinal physiological environment are a major consideration. The gastrointestinal physiological environment and strategies for selecting wall materials are summarized in Figure 1.

### 3.1. Stomach

The intricate physiological environment of the stomach poses specific challenges to the design and use of intestinal-targeted release microcapsules. The acidic nature of gastric juice (pH 1.5–3.5), the presence of pepsin, and the protective function of gastric mucus together establish a unique microenvironment [48]. These factors significantly affect the stability and release properties of microcapsules, guiding the selection and design of their wall materials.

Hydrochloric acid is a major component of the stomach’s environment, and its acidic properties pose a significant challenge to the stability of microencapsulated wall materials. Many natural and synthetic polymers, when used as wall materials, are vulnerable to degradation or solubilization under acidic conditions. This leads to the premature release and degradation of phenols in gastric acid and prevents phenols from reaching the intestines and fully exerting their functional effects. Pepsin, the dominant digestive enzyme in gastric juice, activates and cleaves peptide bonds in proteins, facilitating their breakdown in an acidic environment. The presence of pepsin can degrade certain protein-based wall materials (e.g., gelatin), causing premature release of the core material and inhibiting the active ingredient from reaching the intestines to perform its intended activity [49,50]. To prevent this, acid-resistant and enzyme-resistant materials, such as pectin, chitosan, sodium alginate, and gum arabic, are often chosen to protect the microcapsules from premature degradation in the gastric environment.

### 3.2. Small Intestine

The small intestine, which plays a central role in nutrient absorption, features a dense network of villi and microvilli that dramatically expand its absorptive surface area. Targeted release of phenols and other bioactive compounds in this region increases their bioavailability, ensuring efficient uptake into systemic circulation and enhancing their physiological efficacy [51]. As the longest part of the digestive system, the small intestine facilitates the enzymatic breakdown of macromolecules, enabling efficient nutrient assimilation. The small intestine’s near-neutral pH (6.5–7.5) provides optimal conditions for digestive enzymes, ensuring the efficient hydrolysis of nutrients [52]. Enzymes such as amylase, protease, and lipase secreted by the small intestine catalyze the breakdown of dietary macromolecules, with nutrient absorption occurring through mechanisms including passive diffusion, active transport, and endocytosis [53]. Thus, pH-responsive encapsulation systems are employed to protect phenols from gastric acidity while facilitating their release in the small intestine. In addition, acid-resistant and enzyme-resistant wall materials further improve the precision of intestinal-targeted delivery.

### 3.3. Colon

The colon, as the final segment of the gastrointestinal tract, is mainly responsible for water and electrolyte absorption, vitamin recovery, and the compaction and expulsion of undigested residues. New insights into the colon’s role in host–microbiota crosstalk have identified it as a critical target for therapeutic interventions involving prebiotics, probiotics, and bioactive compounds to promote systemic health [54]. Colon-targeted phenol delivery has been shown to alleviate colitis, modulate gut microbiota, and strengthen the intestinal barrier, offering both therapeutic and preventive benefits [55]. The design of phenol carriers, by taking into consideration the colon’s physiological characteristics, ensures targeted delivery while minimizing unintended effects. The colonic lumen has a mildly alkaline pH (6.5–7.5) [56], which supports both enzymatic and microbial activities. The colon harbors a dense microbial community (10^11^–10^13^ CFU), which greatly exceeds the microbial load in the stomach (10^3^ CFU) and small intestine (10^4^–10^8^CFU) [57]. Obligate anaerobes, including *Bifidobacterium*, *Lactobacillus*, and *Bacteroides* species, predominate in this ecosystem, and their metabolic processes are enhanced by the alkaline pH of the colon. These microorganisms are vital for maintaining gut health, immune function, and metabolic homeostasis [58,59]. Thus, materials that resist degradation in the gastric and small intestinal environments but can be metabolized by colonic microbes are suitable for achieving colon-targeted release. Moreover, the higher pressures in the colon, resulting from peristalsis, may serve as an effective trigger for drug release [60].

Colon-targeted delivery systems require enhanced stability and prolonged intestinal retention compared to those targeting the small intestine. To achieve this, formulations often incorporate a combination of polysaccharides and proteins to strengthen the system’s resistance to the harsh gastric and small intestinal environments. This ensures that the delivery system reaches the colon intact, enabling precise and site-specific release. Such an approach improves both the physiological resilience and release performance of the system in the colonic environment [19].

In summary, the gastrointestinal tract presents compartment-specific physiological environments: an acidic gastric lumen, a near-neutral small intestine, and a microbiota-rich colon. These environments impose distinct design requirements for microencapsulation. In the stomach, low pH (1.5–3.5), pepsin activity, and the mucus barrier can degrade polymer walls and prematurely release phenols. The small intestine (pH 6.5–7.5) necessitates acid-resistant yet pH-sensitive carriers that remain stable in gastric conditions but release contents site-specifically. In the colon, where the pH remains mildly alkaline and microbial density is high, delivery systems must exhibit microbial and enzymatic responsiveness, mechanical robustness, and extended retention. Thus, the selection of wall materials must account for the entire gastrointestinal landscape to ensure successful transit and precise release in the colon, enabling the bioactive payload to perform its intended function.

## 4. Wall Materials for Intestinal-Targeted Release

In the development of intestine-targeted delivery systems, the selected materials must possess sufficient mechanical strength to resist the oral and gastric environments while ensuring controlled release in the small intestine and colon. Therefore, the choice of wall material is of great significance. It is necessary to take into account the physicochemical properties of phenols, such as their hydrophilic/hydrophobic characteristics and charge attributes. In addition, to achieve targeted release in the small intestine or colon, both the mechanical strength and gastrointestinal stability of the wall material must be carefully considered. The following is an introduction to the commonly used wall materials in these two types of systems.

### 4.1. Small Intestine Targeted Release

Optimal wall materials for small intestine-targeted microcapsules must demonstrate high biocompatibility, chemical stability, and resistance to gastric acid. To overcome gastric conditions (pH 1.5–3.5, proteolytic enzymes, bile salts), pH-sensitive polymers—such as alginate, chitosan, and resistant oligosaccharides—are commonly employed to ensure site-specific payload release in the small intestine. Given its central role in nutrient absorption, the small intestine requires well-designed delivery systems to improve the bioavailability and chemical stability of phenols. The physicochemical properties of encapsulating materials critically influence release kinetics across the gastrointestinal tract (oral cavity, stomach, small intestine), ultimately determining therapeutic outcomes. High-performance carriers must withstand degradation in the oral and gastric environments, while enabling pH- and enzyme-responsive release in the small intestine, with minimal leakage into the colon. The subsequent sections discuss commonly used wall materials for phenols encapsulation targeted to the small intestine.

#### 4.1.1. Chitosan

Chitosan, a cationic polysaccharide obtained through chitin deacetylation, demonstrates excellent mechanical strength and resistance to gastrointestinal shear stress [61,62]. Its functional groups (-NH_2_, -OH, -COCH_3_) facilitate non-covalent interactions, including hydrogen bonding and electrostatic attraction with phenols, thereby enhancing payload stability [63]. Under gastric conditions (pH 1.5–3.5), protonation of the amine groups promotes electrostatic crosslinking, while the polymer’s hydrophobic nature further prevents premature drug release [16]. At intestinal pH (7.0–7.5), chitosan undergoes deprotonation, which enables solubilization, while enzymatic degradation by proteases and carbohydrases facilitates site-specific release [54]. The cationic surface charge also promotes mucoadhesion through electrostatic interactions with negatively charged intestinal mucins, extending residence time and increasing local concentrations of phenols, thus improving absorption [16]. Its biocompatibility and low immunogenicity further support chitosan’s potential for clinical application [64]. The dual pH responsiveness—resistance in the stomach and solubility in the intestine—makes chitosan ideal for spatiotemporal delivery systems. Accordingly, chitosan is considered a leading candidate for small intestinal drug delivery. In one example, Hassan et al. [65] developed microencapsulated systems by coating alginate–caffeic acid hydrogel beads with chitosan using ionic gelation. The chitosan layer minimized drug leakage in Simulated Gastric Fluid (SGF) (<20%) via surface erosion and Fickian diffusion, while in Simulated Intestinal Fluid (SIF), over 90% release was achieved within 180 min [65].

#### 4.1.2. Sodium Alginate

Sodium alginate, a natural polysaccharide derived from brown algae such as kelp and Sargassum, is the sodium salt of alginic acid [66]. This biocompatible and non-toxic polymer is extensively applied in food and pharmaceutical delivery systems [67]. Structurally, it consists of β-D-mannuronic acid and α-L-guluronic acid residues linked via (1→4) glycosidic bonds [66]. As a polyanionic polymer, sodium alginate exhibits pH-responsive solubility: in acidic conditions (pH 1.5–3.5), protonation of carboxyl groups reduces hydrophilicity and contracts the polymer chains, contributing to gastric stability. In contrast, in the small intestine (pH 6.0–7.5), deprotonation increases solubility, facilitating pH-dependent release of encapsulated compounds [68]. Its excellent film-forming properties allow the construction of microcapsules with sufficient mechanical strength to withstand oral mastication and gastric peristalsis, thereby protecting sensitive phenols [69]. When crosslinked with divalent cations such as Ca^2+^, sodium alginate forms ionotropic hydrogels that remain intact under gastric pH. These hydrogels disintegrate in the intestinal environment through a combination of cation exchange (Na^+^ replacing Ca^2+^) and pH-induced dissolution, enabling targeted intestinal release [68].

Zhao et al. [70] developed alginate-based enteric capsules to enhance the oral bioavailability of proteins. These capsules maintained structural integrity in SGF (pH 1.2) but disintegrated rapidly in SIF (pH 6.8), demonstrating their effectiveness for site-specific protein delivery [70]. Similarly, Cao et al. [71] reported that sodium alginate-composite microcapsules significantly improved microbial viability under simulated gastrointestinal conditions. *Lactobacillus plantarum* showed a 71.49% survival rate in SGF and was released at 2.51 × 10^9^ CFU in SIF, underscoring the formulation’s resilience during digestion [71]. González et al. [72] optimized low-concentration alginate matrices for sustained intestinal release of olive secoiridoids. The system’s sensitivity to alkaline pH and extended intestinal retention provided gastric protection while enabling controlled release, resulting in 88% bioaccessibility and improved systemic bioavailability [72].

#### 4.1.3. Other Wall Materials

Protein-based encapsulation presents a promising strategy for targeted phenol delivery to the small intestine. Wang et al. [73] developed microcapsules using soy protein isolate (SPI) and whey protein isolate (WPI) as wall materials. These formulations effectively protected curcumin from gastric degradation and facilitated its release in the small intestine through pH and enzyme-responsive mechanisms. Among the two microcapsules, WPI-based microcapsules exhibited higher encapsulation efficiency (97.6%) and payload capacity (9.6%), whereas SPI-based microcapsules demonstrated greater proteolytic sensitivity and a cumulative curcumin release exceeding 85% during intestinal digestion [73].

In conclusion, pH-responsive polymers (e.g., chitosan, sodium alginate) and protein composites (e.g., SPI, WPI) are rationally selected as wall materials to enhance microcapsule stability and achieve site-specific release. These materials synergistically resist gastric acidity and promote controlled intestinal release, thereby improving phenol bioaccessibility and systemic uptake.

### 4.2. Colonic Targeted Release

Phenols have shown therapeutic potential in alleviating colitis and modulating gut microbiota through colon-specific mechanisms [74,75]. These effects are mediated by colonic microbiota, which transform phenols into bioactive metabolites via enzymatic cleavage of complex molecular structures [76]. However, unencapsulated phenols are susceptible to extensive degradation in the upper gastrointestinal tract (GIT), leading to reduced colonic bioavailability and compromised anti-inflammatory and microbiota-regulating efficacy [77,78]. Conventional microencapsulation techniques often fail to withstand gastric and intestinal environments due to inadequate mechanical strength, resulting in premature release of the payload in the upper GIT. This underscores the need for advanced colon-targeted delivery systems with improved gastrointestinal stability.

In designing such systems, physicochemical properties of wall materials need to be considered to guarantee the phenol stability at different GIT environments. Effective colon-targeted carriers must endure sequential physiological stresses—including gastric acidity (pH 1.5–3.5), digestive enzymes (e.g., pepsin, trypsin), and microbial fermentation in the colon. An ideal system should resist degradation throughout the oral cavity, stomach, and small intestine, and enable precise release in the colon to ensure sufficient local concentration for therapeutic activity. To meet these criteria, composite wall materials are commonly employed. Multilayered matrices can enhance structural stability and enable site-specific release in the colon.

#### 4.2.1. Wall Materials for Microbial Degradation

The degradation of encapsulation materials in the colon is largely mediated by resident anaerobic bacteria, including *Bifidobacterium*, *Lactobacillus*, and *Bacteroides*. These microbes can specifically degrade certain polysaccharides, such as pectin and guar gum. Thus, polysaccharides are considered as effective wall materials for colon-targeted delivery. Their microbial metabolism promotes the controlled release of phenols in the lower gastrointestinal tract.

Pectin is a plant-derived heteropolysaccharide, which can form stable phenol encapsulation complexes through hydrogen bonding and electrostatic interactions. Its hydrophobic domains facilitate the selective encapsulation of curcumin, achieving up to 85.3% binding efficiencies [79]. Pectin degradation in the colon requires the synergistic action of microbial enzymes, including hydrolases, lyases, and esterases, produced by colonic microbiota such as *Bacteroides* and *Bifidobacterium* [80]. Pectin is resistant to degradation by endogenous enzymes in the stomach and small intestine. However, enzymes from intestinal microbes (primarily species from *Mycobacterium*, *Bifidobacterium*, and *Clostridium*) can break it down in the colon. This makes pectin a suitable material for colon-targeted phenol delivery [81]. However, pectin’s hydrophilicity and pH-dependent swelling can limit its standalone use. To address these limitations, composite systems like pectin–chitosan polyelectrolytes are used. These systems can reduce aqueous solubility to less than 15% in SGF [82], while enhance stability and enable the controlled release of phenols in the colon [16]. For example, Andishmand et al. [82] developed a chitosan–pectin polyelectrolyte complexes. The complexes retained over 80% resveratrol during simulated upper gastrointestinal transit, and achieved 49% colon-targeted release of resveratrol [82].

Guar gum is a natural polysaccharide primarily extracted from guar beans (*Cyamopsis tetragonolobus*). It consists of mannose and galactose units, and is characterized by high water solubility and biodegradability [83,84]. The hydrophilic matrix of guar gum formed in cold water allows for the encapsulation of phenols with varying solubility profiles [85]. Guar gum exhibits pH-dependent swelling behavior in the gastrointestinal tract: it swells minimally in acidic conditions and undergoes maximal expansion in alkaline environments [86]. Furthermore, guar gum is resistant to digestion in the stomach and small intestine, but can be efficiently degraded by microbes in the colon. Gut microbes that can degrade guar gum include *Bacteroides*, *Ruminococcus*, and *Bifidobacterium* [87,88]. These characteristics make guar gum an effective wall material for colon-targeted phenol delivery. Kumar et al. [89] developed solid tablets using guar gum-encapsulated curcumin. In a simulated gastrointestinal environment over 24 h, these solid tablets released 42–86% of curcumin in the colon, while only 10% of the curcumin was released by the unencapsulated form. In an in vitro cytotoxicity study, the curcumin-encapsulated tablets exhibited stronger anticancer activity against HCT-15 cells than free curcumin, without any cytotoxic effects from the blank matrix. The underlying mechanisms of this enhanced activity warrant further investigation [89].

#### 4.2.2. Reinforcement of Wall Materials to Withstand Small Intestine Digestion

Colon-targeted phenol delivery systems require greater mechanical and chemical robustness than those intended for small intestinal release. By selecting more robust wall materials, the colon-targeted phenol delivery system can effectively withstand small intestine digestion. To facilitate the release of core materials in the colon, Sun et al. [90] engineered polysaccharide-reinforced matrices that improved systemic stability, resulting in 92% colonic payload delivery in murine colitis models. Additionally, colon-targeted phenol release can be achieved by optimizing the concentration and preparation method of the wall materials. González et al. [72] showed that microcapsules of olive bitter glycosides, prepared with a higher concentration of sodium alginate and by spray-drying, overcame the sensitivity of alginate to the alkaline environment of the small intestine and the extended transit time to the colon. This approach prevented premature release of olive bittersweet in the small intestine, ensuring its intact release in the colon (90%) [72].

Through design modifications, wall materials intended for small intestinal-targeted release can be used for colon-targeted delivery. For instance, alginate can provide gastric protection and chitosan can provide small intestinal enzyme resistance. The composite matrices of alginate and chitosan enable precise colonic delivery [16]. Wen et al. demonstrated the pH and enzyme-responsive release of quercetin using alginate–chitosan carriers, achieving 85% colonic bioavailability in pharmacokinetic studies [91].

In summary, for small-intestinal targeted delivery of phenols, a common system involves coating phenols with a large-molecular-weight polysaccharide (e.g., chitosan, sodium alginate) wall material. If the phenols are hydrophobic, a protein or a hydrophobicity-inducing polysaccharide is used as an inner layer. This ensures the system has enough mechanical strength to survive the gastric environment and releases phenols stably in the small intestine. Colonic drug delivery demands greater system strength and stability. To enhance mechanical stability, an additional protective layer can be applied to the small intestine-targeted delivery system. Alternatively, select wall materials degradable by colonic microbes for the inner layer and pair them with other large-molecular-weight polysaccharides as the outer layer. This shields phenolic compounds from degradation in the stomach and small intestine. Sometimes, proteins are added to enhance the solubility and embeddability of hydrophobic phenols.

## 5. Intestinal Release of Phenolic Compounds

**Figure 2:**
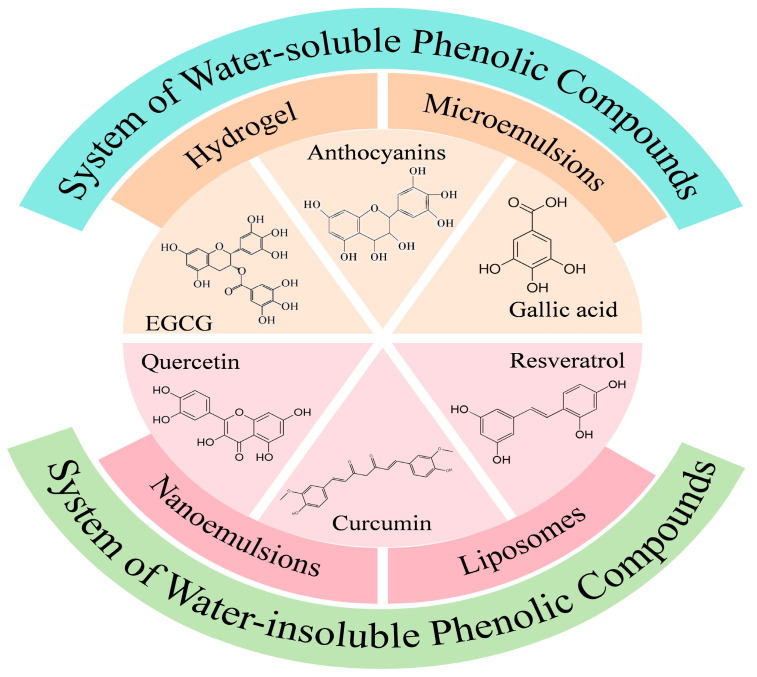
Schematic of delivery systems for water-soluble and non-water-soluble phenolic compounds.

Recent research has made significant progress in developing delivery systems for phenolic compounds. These studies aim to enhance the bioavailability and functionality of phenolic compounds, thereby maximizing their health benefits in the gut. Phenolic compounds such as gallic acid, anthocyanins, quercetin, resveratrol, and curcumin possess unique bioactivities and health benefits. However, these compounds face challenges in intestinal delivery, including low solubility, instability, and poor bioavailability (Table 1). Delivery systems must be carefully selected based on the distinct physicochemical properties of each phenolic compound. Amongst these physicochemical properties, water solubility needs special focus in designing targeted delivery systems of phenolic compounds (Figure 2).

### 5.1. Intestinal Targeted Delivery of Water-Soluble Phenolic Compounds

For water-soluble phenolic compounds such as gallic acid, anthocyanins, and epigallocatechin gallate (EGCG) [92,93], their favorable water solubility facilitates smooth transport through the gastrointestinal tract and rapid dispersion for absorption by the intestinal mucosa. However, these compounds remain susceptible to degradation from gastric acid and digestive enzymes. To overcome this challenge, researchers have developed targeted release systems based on hydrogels and microemulsions.

Hydrogel systems, with their hydrophilic network structure, can effectively load water-soluble phenolic compounds. By adjusting the cross-linking density and pore size of the hydrogel, the release rate of the compounds can be controlled to achieve intestinal targeted delivery [94]. Winda et al. prepared a hydrogel loaded with gallic acid using alginate gel and lactoferrin. The hydrogen bonds formed between the phenolic groups of gallic acid and lactoferrin effectively prevented the loss of gallic acid from the gel, thereby enhancing its stability [95]. Meanwhile, Zhao et al. [96] developed a nano-hydrogel system for water-soluble anthocyanins using alginate and hyaluronic acid as wall materials. This system can target the release of anthocyanins at sites of intestinal inflammation. Utilizing a colon-specific alginate gel shell and hyaluronic acid for inflammation-specific targeting, the nano-composites aggregate on the inflamed colon mucosa. This design protects anthocyanins from degradation in external and gastrointestinal environments and significantly improves local bioavailability by prolonging colon retention time [96,97].

Microemulsions significantly enhance the dispersion of water-soluble phenolic compounds within the gastrointestinal tract, thereby enhancing their bioavailability. Gao et al. [98] utilized a reverse microemulsion process to develop a modified corn starch microgel system. This innovative system achieved an impressive encapsulation efficiency of 63.89% for EGCG. Notably, the DPPH free radical scavenging activity of EGCG was elevated to 85.37%, reflecting the system’s efficacy. Furthermore, the system demonstrated a markedly higher release of EGCG in SIF (60%) compared to SGF (<20%), clearly indicating its intestinal targeted delivery characteristic [98].

### 5.2. Intestinal Targeted Delivery of Water-Insoluble Phenolic Compounds

Quercetin, resveratrol, curcumin, and other phenolic compounds with poor water solubility present significant challenges in terms of gastrointestinal absorption and utilization [99,100,101]. The low solubility of these compounds in the gastrointestinal tract impedes the formation of a uniform solution, thereby limiting their contact with the intestinal mucosa and leading to reduced bioavailability [102,103]. To overcome these challenges, research has been directed toward the development of advanced drug delivery systems designed to enhance the solubility and stability of these compounds. Among the various approaches, nanoemulsions and liposomes have emerged as two of the most commonly utilized targeted release systems for water-insoluble phenolic compounds.

Nanoemulsions significantly enhance compound solubility via their nano-sized oil droplets. The surfactants in nanoemulsions prevent droplet aggregation, thereby ensuring stability within the gastrointestinal tract [104]. Chen et al. developed a whey protein isolate–inulin complex emulsion for quercetin encapsulation, achieving an encapsulation efficiency of 83.62% and remarkably enhancing water solubility by 389 times [105]. This delivery system demonstrated superior antioxidant capacity. Moreover, it maintained stability under gastric conditions, enabling targeted quercetin release in the small intestine and thereby improving bioavailability. Additionally, Wang et al. [106] formulated a dextran-based water-in-oil emulsion loaded with curcumin. This system protected curcumin from degradation and significantly enhanced its gastrointestinal absorption, leading to a 4.8-fold increase in bioavailability. It effectively addressed the challenges of curcumin’s poor water solubility and low oral bioavailability [101,106,107].

Liposomes, with their distinctive bilayer membrane structure, encapsulate water-insoluble phenolic compounds within the lipid bilayer, shielding them from adverse gastrointestinal conditions and facilitating absorption through interaction with intestinal mucosal cells [108,109]. Shruthi et al. [110] developed a resveratrol-encapsulated liposome using maltodextrin, lactose, and branched starch as wall materials. This liposome demonstrated stability in SGF and achieved a targeted release of 78.2% of resveratrol in SIF, thereby enhancing its bioavailability and water solubility [110,111].

In summary, the selection of delivery systems for phenolic compounds with varying water solubilities is a direct reflection of their differing physicochemical properties. Water-soluble phenolic compounds are more effectively delivered through hydrophilic systems such as hydrogels and microemulsions, whereas water-insoluble phenolic compounds are better suited to systems like nanoemulsions and liposomes, which are designed to improve solubility and stability. This classification based on water solubility provides a robust theoretical foundation and practical guidance for the development of precise intestinal targeted delivery systems tailored to the specific characteristics of different phenolic compounds.

## 6. Intestinal Delivery of Phenol-Containing Mixtures

In recent years, research on delivery systems for phenols has evolved from focusing solely on the encapsulation of individual compounds to exploring multi-component co-delivery systems. This transition is driven by the potential of co-delivery systems to simultaneously encapsulate multiple bioactive compounds, enabling synergistic interactions that enhance therapeutic outcomes. Consequently, there has been a growing interest in co-embedding phenols with other bioactive agents for targeted delivery to the gut, particularly when the co-embedded compounds are functionally compatible and capable of producing complementary biological effects [21,22]. Table 2 provides a comprehensive overview of recent studies on the co-encapsulation of polyphenols with other bioactive components, detailing their targeted encapsulation characteristics and the resulting biological activities.

### 6.1. Intestinal Co-Delivery of Multiple Phenols

Studies have demonstrated that combining phenols can enhance their overall bioactivity [112]. EGCG, a principal component of green tea phenols, possesses abundant phenolic hydroxyl groups and exhibits potent antioxidant, anti-inflammatory, and colon cancer-preventive properties. When co-administered with curcumin, EGCG has been shown to synergistically enhance its anticancer efficacy [26,104,113]. Moreover, selecting phenols with complementary physicochemical properties for co-embedding can significantly improve the stability and intestinal targeting of delivery systems. For instance, strongly hydrophobic curcumin can be incorporated into the hydrophobic core of zein–lysozyme nanoparticles via an anti-solvent method, while moderately hydrophobic EGCG can be adsorbed in the interfacial region between the zein core and caseinate shell. Yan et al. [23] leveraged these interactions to develop a novel plant protein-based nanoparticle system for co-encapsulating curcumin and EGCG with differing polarities. This system was achieved through EGCG-mediated covalent bonding with proteins or polysaccharide [23]. Enhanced encapsulation efficiency for curcumin, improved stability and bioavailability, and an increase in the composite’s overall antioxidant capacity was further observed. These findings indicate that co-embedding phenols with distinct polarities can both stabilize protein-based delivery systems and induce synergistic therapeutic effects.

Curcumin and resveratrol exhibit a range of biological activities, including antioxidant, anti-inflammatory, and anticancer effects [114]. As hydrophobic phenols, both of them can interact with hydrophobic domains of proteins. By forming a hydrophobic nanoparticle core via protein–phenol coupling, high-efficiency co-encapsulation of curcumin and resveratrol can be achieved. Based on Yan et al.’s preliminary work [79], Liu et al. [115] designed a colloidal delivery system capable of co-delivering both compounds, which enhanced their water dispersibility, chemical stability, and bioaccessibility. The system achieved encapsulation rates of about 71% for curcumin and 85% for resveratrol. In vitro digestion experiments revealed that the formulation resisted degradation in the gastric environment and released its contents in the small intestine. This behavior was largely attributed to the formation of mixed micelles by lipid digestion products, facilitating solubilization of microcapsules in intestinal fluids and increasing the bioaccessibility of curcumin and resveratrol to 61.1% and 90.1%, respectively [115]. Interestingly, the co-loaded nanoparticles were smaller in size compared to those loaded with individual phenols. This is likely due to the close hydrophobic interactions between curcumin and resveratrol, which reduce internal molecular free space and promote a more compact nanoparticle structure.

### 6.2. Intestinal Co-Delivery of Phenols and Carotenoids

Carotenoids are widely distributed in green vegetables and yellow to orange fruits, with a range of biological activities [116]. Structurally, they can be divided into two main types: hydrocarbon carotenoids (such as α-carotene, β-carotene, γ-carotene, and lycopene) and oxygen-containing carotenoids, which are also known as xanthophylls (such as lutein, zeaxanthin, and astaxanthin) [117]. These compounds are recognized for their antioxidant, immunoregulatory, anticancer, and anti-aging effects [118]. Nevertheless, the extensive unsaturation in their hydrocarbon chains compromises their water solubility and bioavailability [119], and renders them susceptible to degradation. To address these limitations, co-pigmentation with active compounds such as phenols and flavonoids has been employed. These molecules can interact with carotenoids via hydrogen bonds, electrostatic interactions, and van der Waals forces, thereby enhancing their stability and antioxidant capacity [120]. Co-embedding carotenoids with phenols is thus a promising strategy for overcoming bioavailability challenges and amplifying functional benefits.

β-carotene is a natural hydrophobic carotenoid and a vitamin A precursor. β-carotene exhibits strong antioxidant properties, including scavenging free radicals and reactive oxygen and nitrogen species. It plays a crucial role in metabolic and cellular developmental processes [121]. However, its poor solubility in water and susceptibility to oxidation limit its use in dietary supplements [32]. Anthocyanins possess anticancer properties. When combined with β-carotene, the resulting formulation can enhance bioefficacy while reducing chemotherapy-related toxicity and resistance. Shi et al. [122] developed a gut-targeted antioxidant delivery system by co-encapsulating liposoluble β-carotene and water-soluble, positively charged anthocyanins using chitosan and oxidized konjac glucomannan as wall materials. The hydrophobic β-carotene was encapsulated within oxidized konjac glucomannan to form a double emulsion, while the positively charged anthocyanins and chitosan were adsorbed onto its surface. This system exhibited pH-responsive release in the intestine, because chitosan resists gastric acid and deprotonates in the neutral environment of the small intestine, facilitating the release of β-carotene and anthocyanin. This delivery system exhibited synergistic antioxidant activity, increased thermal stability, and successfully delivered both antioxidant compounds to the small intestine [122].

As a type of carotenoid, lutein is mainly obtained from marigold petals [123]. It serves as a key component of the human macular pigment, contributing to improved vision and retinal health [124]. Despite these benefits, lutein exhibits low water solubility and high susceptibility to oxidative, thermal, and photolytic degradation [125]. To address this limitation, Yan et al. [126] developed a lutein-enriched emulsion with enhanced stability. A covalently bonded phenol–protein–polysaccharide complex (bovine serum albumin, chlorogenic acid, and dextran) was used as a novel emulsifier, along with vitamin E as a fat-soluble antioxidant. This formulation significantly improved the bioaccessibility of lutein (62.3%), resisted degradation by gastric acid and pepsin, and allowed for targeted release in the small intestine [126]. The presence of chlorogenic acid contributes to the improved physicochemical stability of the system.

### 6.3. Intestinal Co-Delivery of Phenols and Probiotics

**Figure 3:**
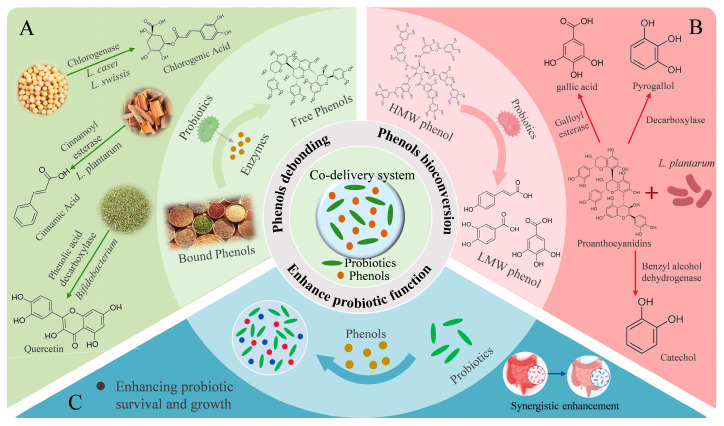
Schematic of the interaction between phenolic compounds and probiotics in co-encapsulation systems. (**A**) Probiotics promote the dissociation of bound phenolics to produce free phenolics. (**B**) Probiotics enhance the biotransformation of phenolic compounds. (**C**) Phenolic compounds promote probiotic growth and have a synergistic effect. *L. casei*: *Lactobacillus casei*; *L. swissis*: *Lactobacillus swissis*; *L. plantarum*: *Lactobacillus plantarum*; HMW phenol: high-molecular-weight phenol; LMW phenol: low-molecular-weight phenol.

Pathogens and harmful substances that are generated during digestion can disturb the microbial balance, and thereby lead to disease. Probiotics have been shown to maintain host health by regulating gut microbiota, modulating immune responses, and enhancing gut barrier integrity [127,128]. Among these probiotics, *Lactobacillus* and *Bifidobacterium* are the most frequently investigated general [129]. Although various single-strain probiotic delivery systems are available [130], Muhammad et al. [131] developed carboxymethylcellulose hydrogels incorporating *Lactobacillus plantarum* along with inulin and soy protein isolate, which significantly improved the storage stability and gastrointestinal viability of the probiotics [131]. However, growing evidence supports the superior efficacy of co-delivery systems that incorporate prebiotics or bioactive compounds, such as phenols [132,133]. These formulations leverage mutualistic interactions: probiotics enzymatically convert phenols into more bioavailable forms, while phenols protect probiotics from hostile environments in the upper gastrointestinal tract [17], enhancing their viability and fostering a beneficial microbiome. The interaction between phenolic compounds and probiotics in co-encapsulation systems are summarized in Figure 3. This synergistic relationship makes co-encapsulation systems a promising approach for colon-targeted delivery, particularly in the alleviation of gut dysbiosis and intestinal inflammation.

#### 6.3.1. Probiotics Promote Phenols Debonding to Enhance Bioactivity

Probiotics can enhance the functional potential of dietary phenols by mediating their enzymatic release from conjugated forms. The rutinose-bound glycosides of flavonols and flavanones are poorly absorbed in the intestine. Notably, *Bifidobacterium* can enzymatically hydrolyze rutin through phenolic acid decarboxylase to release the active aglycone to produce quercetin [134]. In addition, *Lactobacillus acidophilus* and *L. plantarum* produce β-glucosidases that break down flavonoid conjugates, leading to the formation of free phenols. This enzymatic release increases the bioavailability and enhances the health benefits of natural phenols [135,136].

Proanthocyanidins must be released from their food matrix to exert biological effects, a step facilitated by microbial metabolism. Holkema et al. co-encapsulated *Bifidobacterium animalis* with cinnamon extract rich in proanthocyanidins using microencapsulation technology [137]. This approach significantly improved the microbial survival in acidic environments and preserved the antioxidant potential of the phenols. These benefits may result from enzymatic activity of *B. animalis*, which enhances the release and bioavailability of proanthocyanidins in the gut.

Some probiotics can release phenolic compounds from plant cell walls during fermentation. They achieve this by producing specific hydrolytic enzymes such as β-glucosidase, cinnamoyl esterase, phenolic acid decarboxylase, and chlorogenase [138]. As a result, the levels of free phenolic compounds increase. Nelson et al. [38] conducted an experiment using *Lactobacillus casei* and *Lactobacillus swissis* to ferment soybeans. The experiment reached to two outcomes. First, it significantly increased the yield of chlorogenic acid. Second, it enhanced the biological activity of chlorogenic acid. These improvements may be attributed to the enzymatic activity of the probiotics, which promotes the release and bioavailability of phenolic compounds during fermentation [38].

#### 6.3.2. Probiotics Promote Phenols Bioconversion to Enhance Bioactivity

The high molecular weights of phenols limit their gastrointestinal absorption, resulting in poor bioavailability. Probiotics can degrade larger compounds into smaller phenolic acids through specific enzymes, enhancing their absorption and physiological effects. For example, *L. plantarum* exhibits galloyl esterase, decarboxylase, and benzyl alcohol dehydrogenase activities, enabling the stepwise metabolism of grape seed phenols (e.g., proanthocyanidins) into gallic acid, pyrogallol, and catechol [139]. Similarly, *Streptococcus thermophilus* and *L. casei* can convert procyanidins into novel derivatives, including procyanidin isomers, m-coumaric acid, and p-coumaric acid, thereby increasing total antioxidant capacity [35]. Moreover, in a dynamic gastrointestinal simulation, *L. plantarum* facilitated the metabolism of grape phenols, significantly increasing the levels of benzoic acid, gallic acid, syringic acid, and vanillic acid in the transverse and descending colon.

Pereira et al. demonstrated that orange juice enriched with microencapsulated *Bifidobacterium longum* R0175 increased fecal phenolic acids, indicating probiotic-induced transformation of flavanones into bioavailable metabolites (phenolic acids) [140]. Using spray drying, Sharma et al. co-encapsulated *L. casei* with berry phenols, resulting in 72.6% encapsulation efficiency and 63.6% bioaccessibility, driven by probiotic-mediated bioconversion [141].

Zhang et al. [142] constructed a microgel-based delivery platform incorporating a natural phenol–metal framework derived from *Mesona chinensis*. The platform was used to simultaneously deliver phenols and *Akkermansia muciniphila* [142]. The *M. chinensis* polysaccharides stimulated SCFA production, jointly ameliorating acute liver injury via the gut–liver axis. In addition, *A. muciniphila* catalyzed the bioconversion of phenols into bioactive phenolic acids, enhancing antioxidant and anti-inflammatory effects. Notably, the phenols acted as selective nutrients for *A. muciniphila*, promoting its proliferation. This dual-function microgel achieved targeted delivery of live *A. muciniphila* to the gut and facilitated hepatic accumulation of phenols, restoring intestinal balance and mitigating oxidative hepatic damage.

#### 6.3.3. Phenols Enhance Probiotic Function

Phenols play a critical role in stabilizing probiotics, improving their survival in the gastrointestinal tract, and promoting growth and metabolic function. *L. case* is noted for its antihypertensive, antihyperglycemic effects, and its capacity to improve human lipid metabolism [143]. However, its oral delivery is challenged by susceptibility to gastric acid and bile, limiting intestinal colonization. To solve this problem, phenols are selected for co-encapsulation based on their hydrophobic or hydrophilic properties. Ma et al. reported that co-encapsulating hydrophobic phenols with hydrophilic *L. casei* improved the gastrointestinal tolerance and storage stability of strain, and enables its successful release in the intestine [144]. Gaudreau et al. [145] co-encapsulated *L. paracasei* ssp. paracasei with green tea extract phenols in calcium alginate microspheres, resulting in enhanced probiotic survival, improved encapsulation efficiency, and increased free radical scavenging capacity. A pectin–whey protein complex further augmented the bioactivity of the encapsulated compounds under simulated gastrointestinal conditions [145]. Cruz et al. [146] used spray drying to co-encapsulate blackberry anthocyanins with *L. acidophilus* in gum arabic and sodium alginate. The probiotic survival in gastrointestinal simulations was significantly improved [146]. In another study, tannic acid and engineered *Escherichia coli* Nissle 1917 (EcN) were co-encapsulated using self-polymerizable aromatic disulfides and sodium alginate. Tannic acid improved EcN’s resistance to oxidative and inflammatory stress, and the colon-targeted delivery system alleviated DSS-induced colitis in mice by reducing inflammation, repairing the gut barrier, and modulating microbiota composition [147].

In addition to co-encapsulating individual probiotics with phenols for therapy, researchers have explored combining probiotic cross-feeding with phenol co-encapsulation to combat colon cancer. Cross-feeding involves the exchange of metabolic products among microbes to facilitate mutual growth and metabolism. For instance, *Lactobacillus gasseri* converts complex carbohydrates into lactic acid, which *Bifidobacterium bifidum* uses to support its growth. Benito et al. [148] designed a chitosan–alginate microcapsule encapsulating *B. bifidum*, *L. gasseri*, and quercetin. In colon cancer-bearing mice, co-encapsulated *B. bifidum* and *L. gasseri* reduced abnormal crypt foci and adenomas by 45% and 60%, respectively. Adding quercetin further enhanced reductions to 57% and 80%. This demonstrates quercetin’s role in significantly boosting the anticancer efficacy of probiotics [148,149].

**Table 2:** Single and co-encapsulated targeted delivery systems for phenolic compounds.

	Phenols Name	Wall Material	Preparation Method	Intestinal Targeting Performance	Biological Activity	References
Single-encapsulation	Quercetin	Chitosan nanoparticles	Coaxial electrospinning technique	Colon-targeted release	Significant improvement in bioavailability, enhanced anticancer activity (induction of cell apoptosis and oxidative stress).	[91]
		Whey protein isolate, inulin	High-pressure homogenization method	Small intestine- targeted release	Enhanced of the water solubility (389 times), increased of the digestibility (8.59%), enhancing both the stability and bioavailability of quercetin.	[94]
	Resveratrol	Shellac resin ammonium salts	Spray drying technology	Small intestine- targeted release	pH-sensitive, controlled and sustained release of resveratrol in simulated intestinal release experiment, excellent stability, enhanced antioxidant activity (higher radical scavenging activity of DPPH and ABTS compared to pure resveratrol).	[150]
		Pectin, chitosan, polyethylene glycol	Layer-by-layer self-assembly technique	Colon-targeted release	Colon-targeted release 49%, improved bioaccessibility.	[74]
	Anthocyanin	Cyclodextrin	Complexation technology	Small intestine- targeted release	Stable in stomach, released in intestine, promoting growth of beneficial bacteria.	[45]
		Sodium alginate, hyaluronic acid, foodborne nanoparticles	Electrostatic self-assembly method	Colon-targeted release	Colon-targeted release 35.9%, pH-responsive, enhanced bioavailability, gut microbiota modulation, anti-inflammatory properties and bioavailability.	[96]
	Curcumin	Alginate, whey protein isolate, gum arabic	Layer-by-layer self-assembly technique	Small intestine- targeted release	Small intestine-targeted release 84%, improved bioavailability.	[104]
	Epigallocatechin gallate (EGCG)	Chitosan, zein	Antisolvent precipitation method	Colon-targeted release	Higher release efficiency in simulated intestinal fatty environment, the antioxidant activity of EGCG was significantly improved, and the microcapsules with zein had an antioxidant activity four times higher than those without zein.	[151]
	Caffeic acid	Chitosan, sodium alginate	Lonic gelation method	Small intestine- targeted release	Resistant to gastric environment, sustained release in small intestine (up to 180 min).	[56]
	Ferulic acid	Potato protein, pectin	PH-driven self-assembly	Colon-targeted release	Resistant to gastric environment, sustained release in intestine, antioxidant activity.	[150]
Co-encapsulation	Curcumin and EGCG	Zein, caseinate	Antisolvent method	Small intestine- targeted release	EGCG enhanced the dispersibility, encapsulation properties, and antioxidant activity of curcumin, enhanced the stability and bioaccessibility (87.3 ± 2.8%).	[23]
	Phenols (quercetin/ rutin/curcumin/tea polyphenols) and *Lactobacillus casei*	Zein, chitosan	Complex coacervation	Colon-targeted release	Resistant to gastric environment, sustained release in intestine, antioxidant activity, improved bioavailability, and quercetin group probiotic activity significantly increased (1.03 × 10^10^ CFU).	[144]
	Gallic acid and *Lactobacillus*	Pectin, sodium alginate, fu brick tea polysaccharide	Layer-by-layer complex coacervation	Colon-targeted release	Alleviating colitis, improved probiotic survival rate (1 × 10^9^ CFU).	[90]
	Anthocyanins and β-carotene	Chitosan, oxidized konjac glucomannan	Electrostatic adsorption method	Small intestine- targeted release	Improved synergistic antioxidant activity (enhanced thermal stability); reduced various cancers, infectious diseases, obesity, cardiovascular diseases.	[122]
	Proanthocyanidins and *Bifidobacterium animalis*	Chitosan, alginate	Complex coacervation	Colon-targeted release	Enhanced anticancer activity (reduced aberrant crypt foci in mice by 57%).	[137,152]
	Chlorogenic acid and lutein	Bovine serum albumin, dextran	Complex coacervation-freeze-drying technology	Small intestine- targeted release	Resisted gastric acid and pepsin, lutein bioaccessibility significantly improved (62.3%).	[126]
	Coumaric acid and arbutin	Gelatin, polyglyceryl polyricinoleate, sodium chloride	Drop-wise dispersion	Colon-targeted release	Controlled release of arbutin and coumaric acid, improved bioaccessibility.	[153]
	Chlorogenic acid and anthocyanins	Maltodextrin or maltodextrin, carboxymethyl cellulose, gum arabic or xanthan gum	Spray drying technology	Colon-targeted release	Higher retention of anthocyanins in encapsulated wild cherry powder (75%). Encapsulation reduced molecular transformation of anthocyanins and chlorogenic acid during in vitro digestion.	[154]

## 7. Conclusions and Future Perspectives

This review evaluates the influence of gastrointestinal physiological conditions on phenol delivery and introduces suitable wall materials for site-specific release. To achieve targeted release of phenols in the digestive tract, factors such as pH, digestive enzymes, and gut microbiota should be fully considered in delivery system designing. Materials such as chitosan, sodium alginate, and pectin have demonstrated potential in optimizing delivery performance under these varying conditions. Simultaneously, the choice of wall material also should be based on the water solubility of phenolic compounds. Phenolic compounds with good water solubility are suitable for hydrogels and microemulsions, while water-insoluble ones are more appropriate for nanoemulsions and liposomes. Co-encapsulation of phenols with carotenoids or probiotics improves their stability, bioactivity, and synergistic effects. These multi-component delivery platforms not only protect labile compounds from degradation but also improve bioaccessibility through complementary physicochemical properties and probiotic-mediated biotransformations. The integration of probiotics promotes phenol debonding and bioconversion, enhancing phenolic bioavailability, while phenols support probiotic survival and functionality in the harsh gastrointestinal environment. Such reciprocal interactions highlight the potential of co-delivery systems for targeted treatment of intestinal inflammation, oxidative stress, and related chronic diseases.

Moving forward, the development of advanced in vitro and simulated gastrointestinal models that more accurately mimic human physiological and microbial conditions remains essential. Moreover, optimizing the core-to-wall material ratio and clarifying its effect on the release behavior and sustained delivery of phenolic compounds require further systematic investigation. Notably, there is a significant lack of clinical trials addressing the release profiles and therapeutic efficacy of these microencapsulation systems in humans, especially considering inter-individual differences in digestion and metabolism. Future research should focus on designing multifunctional delivery platforms that integrate targeted release, protective encapsulation, and bioresponsive properties to bridge the gap between in vitro and in vivo results. In terms of applications, these delivery systems hold promise as plant-based dietary supplements in tablet or beverage forms aimed at alleviating conditions such as colitis and hepatitis. Such advancements are critical to fully realizing the preventive and therapeutic potential of phenolic compounds in managing chronic diseases.
